# Socioeconomic and demographic profile of occupational morbidity and mortality in Brazil from 2009 to 2016

**DOI:** 10.47626/1679-4435-2021-589

**Published:** 2021-04-30

**Authors:** André Luís de Medeiros Prudêncio, Beatriz Gonçalves Marques, Débora Rodrigues Aguiar, Laís Cruz Lima, Lara Damiani Cabral, Rafaella Willig Quadros, Flavio Ricardo Magajewski

**Affiliations:** 1 Curso de Medicina, Universidade do Sul de Santa Catarina, Tubarão, SC, Brazil

**Keywords:** occupational accident, morbidity, mortality rate

## Abstract

**Introduction:**

Occupational accidents occur as a result of work, and can lead to bodily harm or functional impairments that lead to death, or to the reduction or loss of working capacity. Occupational accidents are associated with two possible outcomes: morbidity or mortality. Morbidity refers to the subset of a population that develops an illness over a given period of time, while mortality refers to the number of individuals who die over a specified time period.

**Objectives:**

To assess occupational morbidity and mortality in Brazil in the period of 2009 to 2016.

**Methods:**

An ecological study was conducted based on secondary data collected from incident records in the Social Security database.

**Results:**

The outcomes of all occupational accidents reported in Brazil from 2009 to 2016 were extracted from the database. These data were then classified by geographical region and category in the National Classification of Economic Activities (Classificação Nacional de Atividades Econômicas), so as to calculate the prevalence of each outcome and the accident mortality rates, and compare these values across regions and occupational categories.

**Conclusions:**

The data show that the outcomes of occupational accidents are directly associated with socioeconomic sectors and the sociocultural characteristics of different regions in the country. These results make an important contribution to the characterization of occupational morbidity and mortality in Brazil.

## INTRODUCTION

According to article 19 in Law No. 8.213/91,^1^

an occupational accident is an event that occurs in the course of employment or as a result of occupational activities described in item VII of article 11 of this same law, and results in physical harm or functional impairment leading to death or the temporary or permanent loss or reduction in occupational functioning [free translation].

Occupational accidents can be classified into three categories: commuting accidents, typical accidents, and occupational diseases. Commuting accidents occur on the way to or from work; typical accidents occur in the course of work; and occupational diseases include both work-related and occupational diseases.^2^ Occupational accidents can have one of two outcomes: morbidity or mortality. In the context of health care and epidemiology, morbidity is a feature of natural communities, and refers to the individuals in a population who become ill (or develop a particular type of illness) in a given period of time. This information is used to analyze the features of diseases and injuries affecting the population. Mortality, on the other hand, is also a feature of natural communities, but refers to the number of individuals who die over a period of time. This metric represents the risk or probability of death within a population or the risk of dying as the result of a particular illness.^3^

Mortality is internationally used as a health status indicator in the assessment and planning of health care policies and programs.^4^ Preventable or reducible deaths are those that could be fully or partially prevented by accessible and effective health care services.^5^ The outcome of occupational accidents, such as morbidity and mortality, can be further classified based on their consequences for workers, which can include the following: need for medical assistance; disability for less than 15 days; disability for 15 days or more; permanent disability or death, all of which are determined by the National Institute of Social Security (Instituto Nacional do Seguro Social; INSS).^2^

These observations underscore the need to explore mortality rates and the prevalence of different forms of disability associated with occupational accidents in Brazil. The aim of this study was therefore to analyze and compare the outcomes of occupational accidents between geographical regions of the country and categories in the National Classification of Economic Activities (Classificação Nacional de Atividades Econômicas; CNAE) 2.0 between 2009 and 2016. This was achieved by collecting secondary data on the morbidity and mortality associated with occupational accidents reported to the Social Security^6^ system from 2009 to 2016; analyzing the mortality rates and prevalence of disability in the workforce; identifying reasons for changes in these rates over time; and comparing the data between different regions in Brazil.

## METHOD

A three-step ecological study was performed to compare the frequency and outcomes of occupational accidents between groups and examine the association between these variables. In the first stage, data were collected from a historical database of occupational accidents available on the Social Security website.^6^ The outcomes of occupational accidents reported in Brazil from 2009 to 2016 were examined and classified by occupational categories in the CNAE 2.0, geographical region and incident type. In a second stage, the data were entered into Microsoft Office Excel 2011 for statistical analysis. The prevalence rate of accidents and outcomes during the study period in each geographical region of the country was calculated by dividing the number of incidents by the mean number of workers. Graphs and tables were then used to provide a better visualization of the results.

Lastly, the results were discussed and used to generate conclusions based on a search of the relevant literature in different databases - SciELO, Google Scholar, Virtual Health Library (VHL), books, theses, legislation, documents and government regulations - in order to analyze the differences between Brazilian regions and the association of these variations to specific regional characteristics. Since the data used in the present study are publicly available in electronic form, this investigation was exempt from ethical review. Furthermore, since the database is anonymous and does not identify individuals or companies, informed consent forms were not required for data collection.

## RESULTS

From 2009 to 2016, across all categories in the CNAE 2.0, a total of 122,937 cases of permanent disability and 21,490 deaths were reported to social security. In the present study, the prevalence of accidents requiring medical assistance was highest among infrastructure construction workers (n = 39,141; 4.60%) (Table 1).

**Table 1:** Occupational morbidity and mortality rates according to the National Classification of Economic Activities (CNAE) 2.0 from 2009 to 2016 in Brazil

CNAE2.0 Category	Medical assistance	Rate*	Disability < 15 days	Rate*	Disability > 15 days	Rate*	Permanent disability	Rate*	Death	Mortality rate
Agriculture, livestock and related services	11,616	0.88	96,041	7.29	60,302	4.58	2,700	0.20	1,261	0.10
Surveillance, security and investigation	2,328	0.39	25,063	4.23	23,923	4.04	1,165	0.20	473	0.08
Wholesale trade, except vehicles	12,873	0.85	91,727	6.08	64,998	4.31	3,113	0.21	1,059	0.07
Retail	16,206	0.26	249,346	4.03	208,376	3.37	7,790	0.13	1,881	0.03
Building construction	12,056	0.97	93,081	7.53	73,841	5.97	4,430	0.36	1,221	0.10
Food manufacturing	65,264	4.39	202,859	13.65	119,284	8.03	5,173	0.35	1,200	0.08
Non-metallic mineral manufacturing	9,936	2.36	41,571	9.88	37,139	8.83	1,979	0.47	541	0.13
Infrastructure construction	39,141	4.60	75,303	8.86	51,358	6.04	2,777	0.33	1,358	0.16
Specialized construction services	8,676	1.19	40,940	5.64	36,068	4.97	2,108	0.29	758	0.10
Land transport	16,812	1.04	100,466	6.20	101,842	6.28	6,506	0.40	2,907	0.18
Total	826,138	2.05	2,584,448	6.40	2,115,280	5.24	122,937	0.30	21,490	0.05

* (number of incidents/mean number of workers in the period) × 100.

The highest prevalence of disability for 15 days or less was in food manufacturing (n = 202,859; 13.65%). The non-metallic mineral manufacturing sector, on the other hand, had the highest frequency of disability for longer than 15 days (n = 37,139; 8.83%), as well as the highest rate of permanent disability (n = 1,979; 0.47%). Lastly, mortality rates were highest in the land transport sector (n = 2,906; 0.18%), followed by infrastructure construction (n = 1,358; 0.16%) (Table 1).

**Figure 1:**
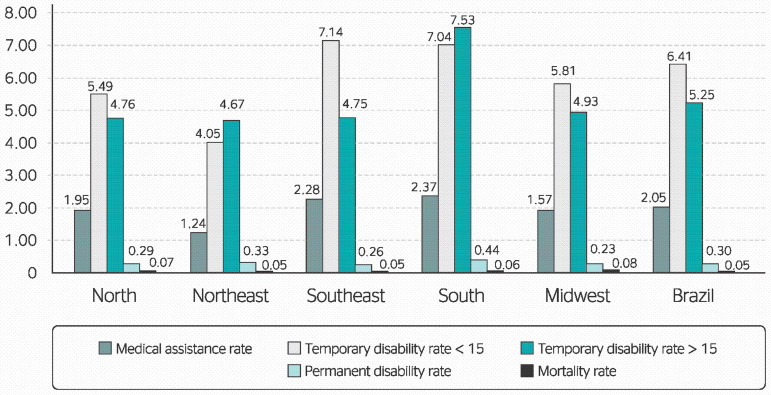
Occupational morbidity and mortality rates per geographical region in Brazil from 2009 to 2016.

As shown in Figure 1, the prevalence of accidents requiring medical assistance was highest in the southern (2.37%) followed by the southeastern (2.28%) region of the country. The frequency of temporary disability (< 15 days) was highest in the Southeast (7.14%), followed by the South (7.04%); and disability periods longer than 15 days were most frequent in the South (7.53%). The South also had the highest rates of permanent disability (0.44%). The comparison of incident outcomes between geographical regions revealed that the highest mortality rates were observed in the Midwest (0.08%) followed by the North (0.07%).

## DISCUSSION

The present study showed that the highest rates of outcomes associated with occupational accidents occurred in southern Brazil. According to a study conducted by Bagolin and Pôrto^7^ this may be attributable to the higher socioeconomic status of the region as well as its university enrolment rate (20.4%).

These factors contribute to a greater awareness of labor rights and standards, and as such, result in higher reporting rates, as illustrated by the information on accident outcomes shown in Figure 1. Furthermore, higher education enrollment rates in northeastern Brazil (7.6%) are significantly lower than observed in the southern region of the country (20.4%) or the national average (14.8%).^7^ These observations, combined with the fact that northeastern Brazil had the lowest number of incident reports in the present study, suggest a connection between socioeconomic status, education level and lower reporting rates.

A separate study noted that, in southern Brazil, the number of incidents for which Accident Report Forms (CAT) had been filed was higher than that of unreported incidents.^8^ These findings corroborate those of the present study, which revealed that southern Brazil had the highest rates of incidents requiring medical assistance (2.37%), temporary disability for 15 days or less (7.53%) and permanent disability (0.44%), as well as the third highest mortality rate (0.06%), all of which may be explained by the high number of reports filed. Research on the distribution of work accidents per category in the CNAE 2.0 revealed that literature on the subject is scarce.^8^ However, the present study provided relevant information on all three sectors of the economy.

Agriculture, livestock and related services had the second lowest mortality (0.10%) and permanent disability rates (0.20%), while the land transport sector had the highest mortality rate (0.18%) and the second highest permanent disability rate (0.40%). This is likely because land transport accidents usually occur in urban areas, where there is better access to health care as well as legal and social services, contributing to the higher reporting rates.^9^ This stands in contrast with rural activities, which have much lower reporting rates since contract workers are not registered in social security. Additionally, the poor oversight of rural workers by state entities lead many employers to ignore safety regulations, and fail to report incidents resulting in death or permanent disability.^10^ As a result, incident rates in agriculture, livestock and related services were lower than those observed in other categories of the CNAE 2.0.

The highest rates of permanent disability were observed in the non-metallic mineral manufacturing sector (0.47%). One example of a non-metallic mineral is fiberglass, whose structure consists of fine glass filaments bound together by silicones, phenols, calcium, aluminum and resins. The latter is considered a carcinogen and a major contributor to atmospheric pollution. In its original form, fiberglass is considered safe, but after treatment with heavy metals such as chromium, it becomes toxic. As such, during the fiberglass production process, workers can be exposed to the material itself or fragments that can irritate the eyes, skin, nose and throat; a high degree of exposure can also aggravate asthma and bronchitis.^11^ All of these factors contribute to the high rates of permanent disability observed in the present study.

The analysis of Table 1, where prevalence rates are divided per CNAE 2.0 category, shows that the retail sector has the lowest rates of incidents requiring medical assistance (0.26%) and accidents resulting in death (0.03%). This is likely attributable to the work environment in the retail sector, which has far fewer risks than the construction sector. The infrastructure sector, on the other hand, was associated with the highest rate of incidents requiring medical assistance (4.60%) and one of the highest mortality rates (0.16%), second only to land transport. These findings reveal that workplace safety and type of occupation have a direct effect on the outcome of occupational accidents.^12^

The present study showed that the outcomes of occupational accidents in Brazil are directly associated with the characteristics of different economic sectors and regional sociocultural variables. The highest mortality rates were observed in the Midwest (0.08%) and the land transport sector (0.18%). These data are crucial for determining the profile of occupational morbidity and mortality in Brazil, so as to increase reporting rates and use the resulting data to improve medical care and reduce the frequency of permanent disability and death. Lastly, the literature search revealed that few authors have examined the consequences of occupational accidents in Brazil, or compared these variables between CNAE categories and regions of the country. There is, as such, a need for further studies on the topic, since these could lead to the implementation of necessary measures that will benefit both employers and employees.
